# Representations of facial features and surface quality in monkey area TE compared to neural network models

**DOI:** 10.3389/fncir.2026.1783892

**Published:** 2026-08-03

**Authors:** Keisuke Shioya, Kazuko Hayashi, Bing Li, Narihisa Matsumoto, Keiji Matsuda, Kenichiro Miura, Mark A. G. Eldridge, Richard C. Saunders, Barry J. Richmond, Yuji Nagai, Naohisa Miyakawa, Takafumi Minamimoto, Shun Katakami, Masato Okada, Kenji Kawano, Yasuko Sugase-Miyamoto

**Affiliations:** 1 Graduate School of Frontier Sciences, The University of Tokyo, Kashiwa-shi, Japan; 2 National Institute of Advanced Industrial Science and Technology, Tsukuba-shi, Japan; 3 Research Fellowship for Young Scientists, Japan Society for the Promotion of Science, Chiyoda-ku, Japan; 4 Section on Neural Coding and Computation, National Institute of Mental Health, National Institute of Health, Bethesda, MD, United States; 5 Institute for the Advanced Study of Human Biology, Kyoto University, Kyoto-shi, Japan; 6 Biosciences Institute, Newcastle University, Newcastle upon Tyne, United Kingdom; 7 Advanced Neuroimaging Center, National Institutes for Quantum Science and Technology, Chiba-shi, Japan; 8 Department of Physiology, Dokkyo Medical University School of Medicine, Mibu, Japan

**Keywords:** facial information processing, temporal visual cortex, neural network models, representation similarity analysis, texture bias

## Abstract

The shape and spacing of facial features are essential for the perception of emotional expressions and the identification of individuals. The surface quality of faces, such as skin texture, eye twinkle, and hair gloss, is valuable for estimating health status. A previous fMRI study in monkeys revealed that regions selective for faces overlapped regions selective for object gloss in the central inferior temporal (IT) cortex, suggesting that the surface quality of faces is processed in area TE. However, the representation of facial surface quality has not been directly examined in this area. To understand neural processing of surface quality in face images, neuronal activity was recorded in area TE of three monkeys while face images with different expressions, identities, and surface qualities were presented. The surface qualities included gloss modulations - high-gloss and low-gloss, and “style-transfer” where facial texture was replaced with fabric. These representations were compared to those in several models, including Convolutional Neural Networks and Vision Transformer. Our results revealed that the style-transferred face images strongly modulated activity in TE neurons and that some neurons were tuned to monkey facial expressions, whereas others were more influenced by surface quality. In contrast, the models showed relatively large contributions of surface-quality representation, particularly to the separation between the original and style-transferred faces, compared with those of facial expression and identity. This trend was observed even in models trained on the stylized ImageNet dataset, which was designed to reduce reliance on texture-based classification. These results suggest a strong influence of image style transfer on both TE neurons and models, the diversity of the facial representations by TE neurons, and the tendency of models to emphasize the style-transferred images regardless of training. These findings highlight a potential limitation in current artificial vision models and underscore the value of examining biological representations to inspire more robust and adaptable visual recognition in future model designs.

## 1 Introduction

The surface quality of a face provides important information, such as in assessing health status. In the ventral visual pathway of monkeys, representations of surface qualities of objects such as gloss have been observed in areas V2, V4, and the inferior temporal (IT) cortex (areas TE and TEO) (Okazawa et al., 2012, 2015, 2017; Goda et al., 2014; Ziemba et al., 2016; Kim et al., 2019). In the IT cortex, the object gloss is processed in detail, including specular and diffuse reflectance (Nishio et al., 2012, 2014). In addition, IT neurons have been reported to be sensitive to multiple surface features from natural objects (Tamura et al., 2016). Meanwhile, numerous studies have shown that neurons in IT cortex respond to images of faces and that facial expressions or identities are represented in their responses (Gross et al., 1972; Yamane et al., 1988; Hasselmo et al., 1989; Sugase et al., 1999; Tsao et al., 2008; Morin et al., 2015; Chang and Tsao, 2017). A functional magnetic resonance imaging (fMRI) study suggests partial overlap between face-selective regions and those representing object gloss in the central IT cortex (Okazawa et al., 2012), suggesting that the surface quality of faces is processed in area TE (area TE includes anterior and central IT cortex, Conway et al., 2010). In the posterior IT cortex, it was also reported that face regions contributed, to some extent, to representation of material properties of objects (Goda et al., 2014). It remains unclear whether neurons in area TE represent facial features (expression and identity) and surface quality, or whether neurons in different regions of area TE prioritize representations of surface quality over representations of facial features.

Recently, significant advancements have been made in neural network models for image recognition, with convolutional neural networks (CNNs) and Vision Transformers (ViTs) becoming some of the most widely utilized models in computer vision. Geirhos et al. (2018) and Tuli et al. (2021) demonstrated that both CNNs and ViTs tend to classify conflict images–where texture and shape associate with different image categories–based more on texture than shape, compared to humans. However, it remains unclear how facial expression, identity, and surface quality are represented within the internal feature spaces of these models. Comparing how TE neurons and neural network models represent stimuli that vary in both texture and shape is valuable for understanding neural mechanisms underlying face perception in the primate visual system and for enhancing neural network models. Thus, we recorded neuronal activity in TE using microelectrodes while presenting faces with different expressions, identities, and surface qualities. This study explored structural differences rather than output differences by contrasting neuronal activity in TE with feature vector in neural network models. Additionally, surface qualities were tested using two qualitatively different modifications: gloss modulations (high-gloss and low-gloss) and a style transfer. The gloss modulations involved changes in local reflective properties of faces (Boyadzhiev et al., 2015), while the style transfer significantly altered the overall texture pattern and was unnatural for faces (Matsuo and Yanai, 2016). It is not yet known how the gloss modifications and style transfer upon face images influence representations by TE neurons. Furthermore, by examining the feature representations in neural network models using the same image sets, this study provides deeper insights into how surface quality is encoded in models and TE neurons.

## 2 Materials and methods

### 2.1 Subjects

The experiments involved two male rhesus monkeys (*Macaca mulatta*; Monkeys A and C, 12 and 6 years old, 10 and 10 kg) and one male Japanese monkey (*Macaca fuscata*; monkey B, 12 years old, 9 kg). For Monkeys A and B, all experimental procedures were approved by the Animal Care and Use Committee of the National Institute of Advanced Industrial Science and Technology (Japan) and were implemented in accordance with the “Guidelines for Proper Conduct of Animal Experiments” (Science Council of Japan) and “Guidelines for the Care and Use of Nonhuman Primates in Neuroscience Research” (The Japan Neuroscience Society^1^). For Monkey C, all experimental procedures were performed under an Animal Study Proposal approved by the Animal Care and Use Committee of the National Institute of Mental Health (USA) and conformed to the Institute of Medicine Guide for the Care and Use of Laboratory Animals. The monkeys were housed in adjoining individual cages that allowed for social interaction with other monkeys as well as interaction with human caretakers.

### 2.2 Surgeries

Surgical procedures of fitting a chamber for chronic recording of single units in Monkey A have been described previously (Sugase-Miyamoto et al., 2014), and those of electrode implantations in Monkeys B and C have been described previously (Mitz et al., 2017; Pearl et al., 2024). Briefly, each monkey first received a surgery of fitting a titanium head holding device to the skull to allow the head to be fixed during the experiments. Then, after behavioral training of the fixation task, a recording cylinder (Crist Instrument Co., Hagerstown, MD, USA) was fitted on the skull over the right hemisphere in Monkey A. Three microelectrode arrays (Utah arrays, iridium oxide, 96 electrodes, 10 × 10 layout, 400 μm pitch, 1.5 mm depth, Blackrock Microsystems, Salt Lake City, UT, USA) were implanted in the anterior part of TE (anterior TE) between the superior temporal sulcus (STS) and the anterior middle temporal sulcus (AMTS) in the left hemisphere of Monkey B (Supplementary Figure 1). One microelectrode array (the identical specifications for both Monkeys B and C) was implanted in the posterior part of TE (posterior TE), i.e., just anterior to the posterior middle temporal sulcus (PMTS) and ventral to STS in the left hemisphere of Monkey C (Supplementary Figure 1). MRIs at 3 T were obtained to determine placement of the head holder and recording chamber (Monkey A) or placement of the head holder and arrays (Monkeys B and C) before the surgeries. MRI scans were also obtained to localize the recording sites in the ventral bank of STS (STSv) in anterior TE for Monkey A (Supplementary Figure 1; Saunders et al., 1990).

### 2.3 Experimental apparatus and behavioral task

The monkeys sat in a primate chair, facing the display screen at a distance of 57 cm from their eyes (the display for Monkeys A and B: GDM-F520, Sony Corporation, Tokyo, Japan; and for Monkey C: 2233RZ 2233RZ, Samsung Electronics, Suwon, South Korea). During behavioral testing, the heads of monkeys A and C were fixed using a head holder, while a head holder and a thermoplastic mask were used for Monkey B. Their gaze was tracked using an infrared pupil-position monitoring system (iRec, AIST, Tsukuba, Japan^2^) (Matsuda et al., 2017). Task control was managed by the REX real-time data acquisition program running on a QNX operating system (Hays et al., 1982). Test stimuli were presented using the Presentation^®^ software (Neurobehavioral Systems, Albany, CA, USA) for Monkeys A and C, and Matlab (The MathWorks, Natick, MA, USA) Psychtoolbox (Kleiner et al., 2007) for Monkey B, all within a Windows operating system (Microsoft Corporation, Redmond, WA, USA).

In the fixation task, the monkeys were trained to fixate on the center of the display while test stimuli were presented. Details of the fixation task have been described in Sugase-Miyamoto et al. (2014), but five images were sequentially presented in this experiment. Each trial began by displaying a fixation point (0.4 degrees in visual angle) at the center of the screen, and the trial started once the monkey’s gaze entered the fixation window (Monkey A: 4 × 4 degrees in visual angle, Monkey B: 4 × 6 degrees, Monkey C: 6 × 6 degrees). In each trial, five images (12 × 12 degrees) were randomly selected from 80 test stimuli (described in the next section) and displayed sequentially at the center of the screen for 350–400 ms each, with an interstimulus interval of 350–400 ms. The fixation point remained visible throughout the trial. If the monkey’s gaze left the fixation window during the image presentation, the trial was immediately terminated, and the same sequence of the five images was repeated in the next attempt. If the monkey successfully maintained fixation within the window during the image presentation and viewed all five images, it received a liquid reward, and the trial was considered valid. One-second inter-trial interval was provided before the next trial began.

### 2.4 Recording and spike sorting

Neuronal activities were recorded from area TE using single tungsten microelectrodes in Monkey A or the microelectrode arrays in Monkeys B and C. In Monkey A, to record single units, a tungsten electrode (Micro Probe Inc., Gaithersburg, MD, USA; Frederic Haer Co., Bowdoin, ME, USA) was vertically inserted through a guide cannula placed in a grid (Crist Instrument Co.) for each recording session, and the electrode was advanced toward area TE using a hydraulic microdrive (MO-97A-S, Narishige Co., Tokyo, Japan). The data from Monkey C consisted of units over three recording days (2021/10/15, 2021/10/21, and 2021/10/27). All recordings were made while the monkeys performed the fixation task. Neural activity data, task event timings, and eye positions were recorded using the REX system for monkey A, the Cerebus system (Blackrock Microsystems, Salt Lake City, UT, USA) for monkey B, and the Grapevine System (Ripple, Salt Lake City, UT, USA) for monkey C. For Monkey A, single units were initially isolated online using either a threshold with dual time-amplitude windows (DDIS-1, Bak Electronics, Germantown, MD, USA) or a spike sorter (Sankei, Tokyo, Japan) based on principal component analysis (PCA) (Abeles and Goldstein, 1977). Unit activities were converted to pulses and recorded at a 1 ms time resolution. For Monkeys B and C, neural signals were digitized (30 kHz) after passing through a band-pass filter (0.25–7.5 kHz). Before each recording session, an initial online sorting was performed by applying thresholds and time-amplitude windows to the signals from each electrode. Subsequently, single-unit spike waveforms were further refined offline using manual inspection, with projections based on PCA in Offline Sorter (Plexon Inc., Dallas, TX, USA).

### 2.5 Test stimuli

**FIGURE 1:**
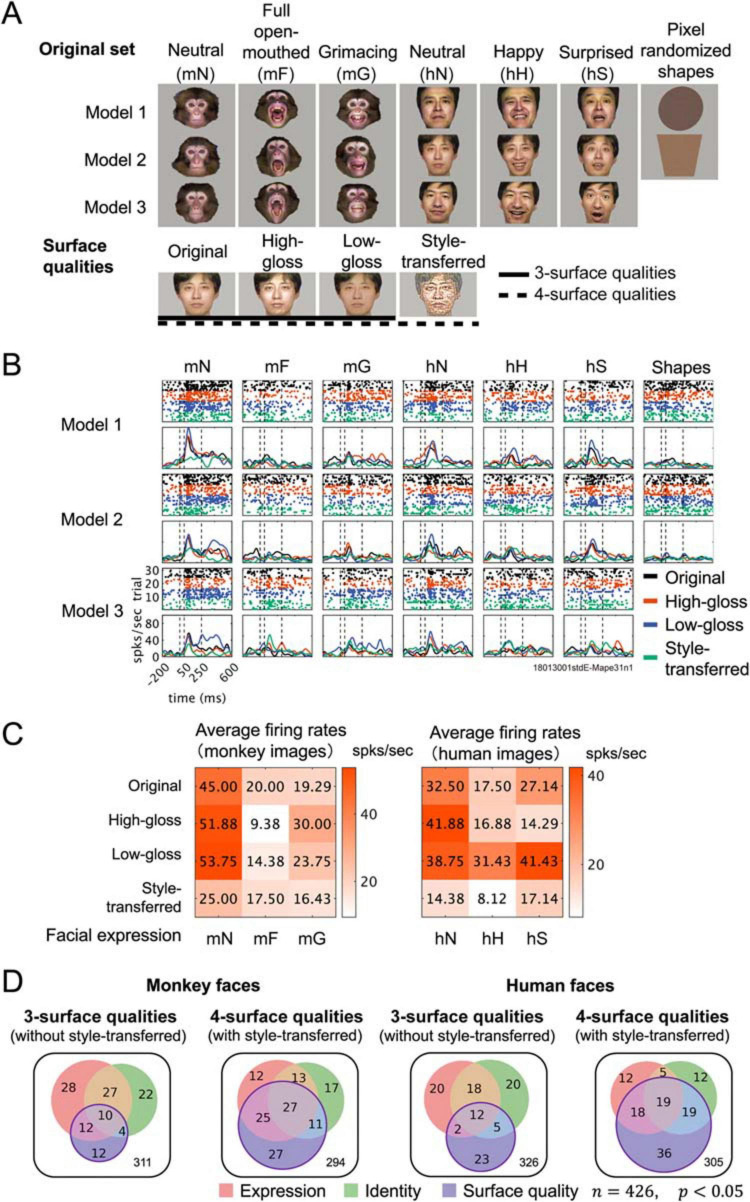
Visual stimuli and neuronal activity in area TE. **(A)** Test stimuli used in the experiment. The top row shows 20 original stimuli (18 faces and 2 shapes). A total of 80 images were created by applying high-gloss, low-gloss, and style-transferred surface qualities. **(B)** Response of a neuron in TE of Monkey B that is selective to the neutral monkey face. The plot positions correspond to the positions of the original set of 20 stimuli shown in panel **(A)**. Each raster plot shows spike times (dots) per trial (vertical axis) over time (horizontal axis), with the line plot indicating the estimated firing rate (spikes/sec), computed using a Gaussian kernel (20 ms SD) and normalized per trial. **(C)** Heatmaps summarizing the average firing rates of the same neuron shown in panel **(B)**, measured within a 50–249 ms window after stimulus onset. Responses are shown separately for monkey and human faces, categorized by expression (monkey: N - Neutral, F - Full open-mouthed, G - Grimacing; human: N - Neutral, H - Happy, S - Surprised) and surface quality (original, high-gloss, low-gloss, style-transferred). **(D)** Venn diagrams showing the number of TE neurons with significant activity changes for each factor (expression, identity, and surface quality) based on a three-way ANOVA (*p* < 0.05). Results are shown for four conditions: monkey faces with 3-surface qualities, monkey faces with 4-surface qualities, human faces with 3-surface qualities, and human faces with 4-surface qualities (original, high-gloss, low-gloss) and 4-surface qualities (including style-transferred). Interaction effects are not represented. For monkey faces, the number of neurons with significant surface-quality effects increased from 38 to 90 with the inclusion of style-transferred images. For human faces, the number increased from 42 to 92. These increases were statistically significant [chi-square test on 2 × 2 contingency tables; monkey face: χ^2^(1) = 35.81, *p* = 2.17 × 10^−9^; human face: χ^2^(1) = 33.83, *p* = 6.01 × 10^−9^], indicating a strong effect of the style-transferred images on TE neuronal activity. Power analyses (G*Power) confirmed comparable sensitivity between the 3-surface quality condition (0.9977) and 4-surface quality condition (0.9954).

The test stimuli consisted of 20 original images: 9 monkey face images [3 models with 3 expressions: neutral (mN), full open-mouthed (mF), grimacing (mG)], 9 human face images [3 models with 3 expressions: neutral (hN), happy (hH), surprised (hS)], and 2 pixel-randomized shapes, an ellipse and a trapezoid. The luminance of the original set was adjusted using the hist match option in the SHINE toolbox (Willenbockel et al., 2010), based on the 9 monkey face images or the 9 human face images. The outline of the ellipse was approximated from the contours of nine monkey images. The pixels of each monkey image were randomized to create nine ellipses, and then those nine ellipses were averaged to create a single ellipse. The pixel-randomized trapezoid was created using the nine human images in the same manner. The size of the test stimuli was around 12° × 12°. From the original set, we created a total of 80 images by applying high-gloss, low-gloss, or style-transferred effects (see Figure 1A). High-gloss and low-gloss images were generated by modifying the HHP (high spatial frequency, high amplitude, positive sign) and LAP (low spatial frequency, all amplitude, positive sign) coefficients according to the method by Boyadzhiev et al. (2015). For the high-gloss images, HHP and LAP were set to [1.50, 0.51], while for the low-gloss images, HHP and LAP were set to [−3.00, −3.00]. Style-transferred effects were performed using a CNN-based method that replaced image textures with fabric patterns while preserving the original colors, with reference to Matsuo and Yanai (2016). Each test stimulus was presented multiple times across recording sessions. The number of trials per test stimulus varied by monkey and session: for Monkey A, the median number of trials per stimulus was 20.0 (interquartile range: 11.0–22.0); for Monkey B, the median was 8.0 (IQR: 7.0–8.0); and for Monkey C, the median was 5.0 (IQR: 5.0–5.0) on each of the three recording days (2021/10/15, 2021/10/21, and 2021/10/27).

### 2.6 Firing rate calculation of TE neurons

For the three-way ANOVA, the mean firing rate within the 50–249 ms time window after stimulus onset was computed for each trial and used as an individual observation. For population-level analyses, including t-distributed stochastic neighbor embedding (t-SNE), dissimilarity matrix construction, Euclidean distance calculation, and centroid distance analysis, the mean firing rates were averaged across all valid trials for each stimulus. The population activity vector for each stimulus was constructed from the mean firing rates of neurons.

### 2.7 Model acquisition

For obtaining the embeddings from neural networks, we primarily used models pre-trained on ImageNet 1k, available through “torchvision.models” in PyTorch (Meta AI, Menlo Park, CA, USA) (torch: 2.5.1, torchvision: 0.20.1). Specifically, we used the following models:

torchvision.models.alexnet(pretrained = True)

torchvision.models.vgg16(pretrained = True)

torchvision.models.resnet50(pretrained = True)

torchvision.models.vit_b_16(pretrained = True)

These correspond to AlexNet (Krizhevsky et al., 2017), VGG16 (Simonyan and Zisserman, 2014), ResNet50 (He et al., 2016), Vision Transformer (ViT) (Dosovitskiy et al., 2020), representing CNNs with increasing depth and architectural complexity, as well as a transformer-based vision model. Additionally, for stylized ImageNet (SIN)-trained neural network models (Geirhos et al., 2018), we used the AlexNet, VGG16, and ResNet50 models provided on GitHub^3^. SIN is a dataset created by applying AdaIN style transfer (Huang and Belongie, 2017) to each image in the ImageNet dataset, randomly transforming each image into one of 79,434 painting styles. In these models, the images retain their original labels based on the objects in the images, despite their style transfer. Therefore, we hypothesized that SIN-trained models would become less sensitive to image modifications using style transfer methods and instead focus more on object features.

### 2.8 Embedding extraction

Matsumoto et al. (2021) reported that, in AlexNet, fully connected layers showed a closer correspondence to area TE than convolutional layers. Based on this observation, we used fully connected output-stage representations for comparison with TE. To ensure consistency across architectures, we extracted representations from the final fully connected layer for all models. Specifically, we used the final fully connected layer (1000 units) for AlexNet, VGG16, ResNet50, and ViT. The same layer selection was applied to the corresponding SIN-trained models. Since the images input to the ViT need to be square, we extended the length of the shorter side of the test stimuli to match the longer side by adding the background color, making them square. For models trained on SIN, the same final layer was used as in the corresponding ImageNet pre-trained models.

### 2.9 t-SNE visualization, dissimilarity matrix and distance calculation between original and surface-quality-modified images

To visualize the representation of test stimuli in the TE neurons and artificial neural networks, we used a method t-SNE (van der Maaten and Hinton, 2008). t-SNE projects high-dimensional data into a low-dimension, making it suitable for visualizing the complex representation in higher visual cortex as it can reflect nonlinear relationships between data points. Liu and Vinck (2022) improved the visualization performance of t-SNE, especially for representation of neuronal populations and neural network models, by applying a transformation called DoD to the Euclidean distance. Therefore, in this analysis, we used t-SNE with DoD transformation for visualization. Because t-SNE primarily preserves local neighborhood relationships and may distort global distances, we did not use the coordinates or distances in the t-SNE embedding for quantitative evaluation. The t-SNE plots were primarily used for visualization and were interpreted alongside subsequent analyses, including DoD-transformed dissimilarity matrices and distance analyses computed in the original full-dimensional representational space.

We calculated pairwise DoD-transformed distances of the images and created a dissimilarity matrix (Kriegeskorte et al., 2008; Yamins et al., 2014). This matrix allows for the visualization and comparison of how different images are represented in the neural populations and models.

To compare the distances between the original and surface-quality-modified face images, Euclidean distance between population activity vectors was calculated for each original high-gloss pair (9 pairs of monkeys and 9 pairs of humans), for each original low-gloss pair (9 pairs of monkeys and 9 pairs of humans), and for each original style-transferred pair (9 pairs of monkeys and 9 pairs of humans) in the original full-dimensional representational space. The three distances from each original image were normalized by dividing each by the sum of the distances. The same procedure was applied to model-derived feature representations. For each stimulus, feature vectors were extracted from the final fully connected layer and flattened to form a vector. Euclidean distances between these vectors were computed for each original high-gloss pair, for each original low-gloss pair, and for each original style-transferred pair, then were normalized.

### 2.10 Centroid distance calculation

To quantitatively compare the impact of differences in expression, identity, and surface-quality on embeddings, we used centroid distance as our metric due to its robustness against outliers. Centroid distances were computed in the original full-dimensional representational space, rather than in the t-SNE embedding. For the 3-surface quality condition (original, high-gloss, and low-gloss), 27 images of monkeys and 27 images of humans were grouped into expression groups (3 groups with 9 images each), identity groups (3 groups with 9 images each), and surface-quality groups (3 groups with 9 images each). Similarly, for the 4-surface quality condition (adding style-transferred), 36 images of monkeys and 36 images of humans were divided into expression groups (3 groups with 12 images each), identity groups (3 groups with 12 images each), and surface-quality groups (4 groups with 9 images each). The centroid of each group was calculated, and the distance between pair of the centroids of each group was measured, and the distances were averaged. For instance, in the case of monkey expression under the 3-surface quality condition, the 27 images were divided into three expression groups based on facial expression: Neutral (mN), Full open-mouthed (mF), and Grimacing (mG), each containing 9 images (3 identities × 3 surface qualities). The centroid of each expression group (mN, mF, mG) was computed, and the three pairwise distances–between mN and mF, mF and mG, and mG and mN–were calculated. These three distances were then averaged to represent the mean centroid distance for the expression grouping. To ensure comparability across different representational spaces, the sum of centroid distances for expression, identity, and surface-quality was normalized to 1 for each model, and the ratios were examined.

### 2.11 Translation to English

The drafting of this manuscript was facilitated using ChatGPT (OpenAI, San Francisco, CA, USA), a large language model (LLM) developed by OpenAI. The API of ChatGPT was utilized to assist in creating natural and concise English expressions, while ensuring the use of consistent terminology throughout the document. ChatGPT was not involved in the authorship or the development of the research content, and it was only used as a tool for translation. The translation process and the resulting text were thoroughly reviewed and validated by the authors to ensure accuracy and adherence to the original content.

## 3 Results

### 3.1 Neuronal responses to face images with different surface qualities in area TE

Neuronal activity in area TE (TE) was recorded from 50 units using microelectrodes (Monkey A), from 219 units using electrode arrays (18 in anterior, 79 in middle, and 122 in posterior of the anterior part of TE) (Monkey B), and from 157 units using an electrode array (Monkey C) while the monkeys performed a fixation task (Methods). The test stimuli included face images with different identities (models), expressions, and surface qualities (Figure 1A). Neurons responded with a variety of selectivity profiles. For example, one neuron responded strongly to the neutral face of monkey model 1 (Figure 1B, identity: model 1, expression: mN). It also showed strong responses to the neutral faces of other monkeys and humans, compared to the shape stimulus, indicating that it is a face-responsive neuron as reported in previous studies (Figure 1B). Regarding the effect of the surface qualities, responses to the high- or low-gloss faces changed compared to the responses to the original faces, whereas the responses to the style-transferred faces were small (Figures 1B,C). Since the difference between the responses to the style-transferred faces and those to the original faces was large, the effect of the surface qualities and facial features were examined for each of the total 426 units using three-way ANOVAs (factors: expression, identity, and surface quality; *p* < 0.05) under two conditions: 3-surface quality condition (levels: original, high-gloss, and low-gloss) and 4-surface quality condition (levels: original, high-gloss, low-gloss, and style-transferred). Figure 1D shows the number of units exhibiting significant effects (*p* < 0.05) for each factor using Venn diagrams. The inclusion of style-transferred images markedly increased the number of TE neurons showing significant effects of surface quality for both monkey and human faces (Figure 1D; chi-square test on 2 × 2 contingency tables; monkey face: χ^2^(1) = 35.81, *p* = 2.17 × 10^–9^; human face: χ^2^(1) = 33.83, *p* = 6.01 × 10^–9^), demonstrating that style-transferred images strongly modulated neuronal activity in TE.

### 3.2 Representation of the test stimuli by TE neurons and neural network models

**FIGURE 2:**
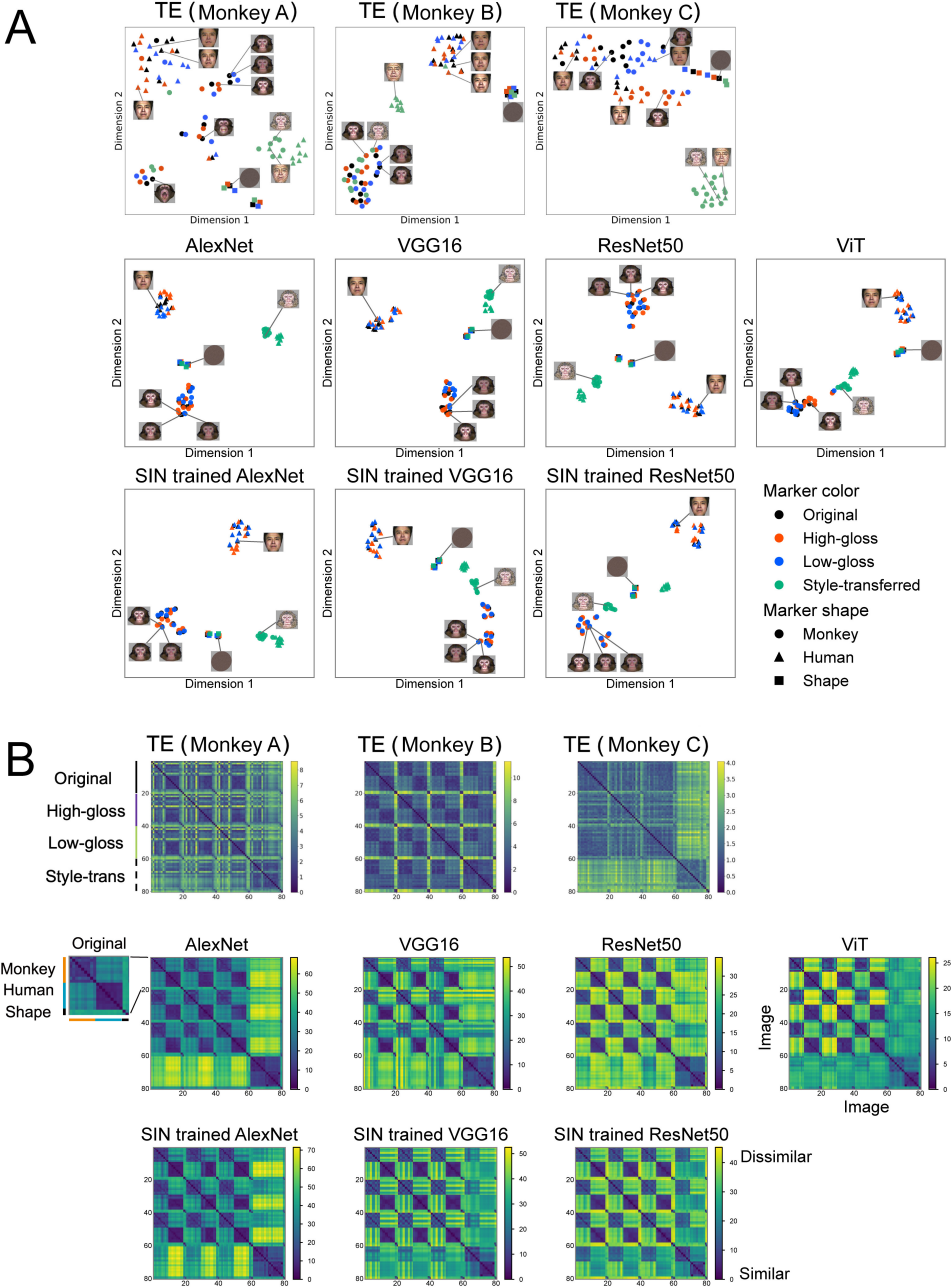
Neural representations in TE neurons and models. **(A)** Representational structure of the test stimuli visualized by DoD-transformed t-SNE. The plots show image representations of the neural populations in area TE of Monkeys A, B, and C, and the feature vectors of the AlexNet, VGG16, ResNet50, ViT, SIN-trained AlexNet, SIN-trained VGG16, and SIN-trained ResNet50 models. Marker color represents surface quality, and marker shape represents the global category (monkey, human, and shapes). Some points are displayed with actual images for clarity. **(B)** Dissimilarity matrices of image representations in the same neural populations and models as in panel **(A)**. The vertical and horizontal axes correspond to images 1–80 (1–20: original; 21–40: high-gloss, 41–60: low-gloss, 61–80: style-transferred images. 1–3: expressions mN, mF, and mG of monkey model 1; 4–6: those of monkey model 2; 7–9: those of monkey model 3; 10–12: expressions hN, hH, and hS of human model 1; 13–15: those of human model 2; 16–18: those of human model 3; 19: an ellipse; 20: a trapezoid). The color at each intersection represents the dissimilarity of the image pair, with blue indicating high similarity and yellow indicating low similarity.

Representations of the test stimuli in both the TE neurons and neural network models were first compared using t-SNE to visualize differences in the encoding of surface quality, expression, and identity. ImageNet-trained models (AlexNet, VGG16, ResNet50, and ViT), along with their SIN-trained counterparts (AlexNet, VGG16, and ResNet50 trained on SIN), were used as the neural network models (see Methods). SIN-trained models were included to examine whether training with stylized images altered the representations of the test stimuli. In TE neurons (Figure 2A, top low), the style-transferred face images were separated from the remaining images. In the first two dimensional space, the style-transferred images of both monkey and human faces were separated in Monkeys A and C, and those of human faces were separated in Monkey B. Aside from the style-transferred faces, the test stimuli were separated by global category (i.e., monkey vs. human vs. shape) in Monkeys A and B. In Monkey A, the representations of monkey faces separated further depending on their expressions. In Monkey C, faces (both monkey and human) and shapes were separated, and many of the high-gloss images seemed to be separated within the faces. In contrast, representations were relatively similar across the models (AlexNet, VGG16, ResNet50, ViT). The style-transferred images of the monkey and human faces were separated from the remaining images, and the separation between monkey faces, human faces, and shapes was observed. In SIN-trained models, similar representations were again observed, the style-transferred face images being separated from the other face images, suggesting that the surface quality, especially the style transfer, has a strong influence on the representation structure, even in these models.

The dissimilarity matrix calculated using DoD-transformed pairwise distances of the test stimuli also revealed that, overall, the style-transferred faces exhibited a distinct pattern compared to the original, high-gloss, and low-gloss faces in both TE neurons and models (Figure 2B). As suggested by the t-SNE visualization, for the original, high-gloss, and low-gloss images, TE neurons showed dissimilarity among monkey facial expressions in Monkey A; global categorization in Monkey B; and in Monkey C, no clear separation between monkey and human faces, but slight dissimilarity between high-gloss and original/low-gloss faces.

**FIGURE 3:**
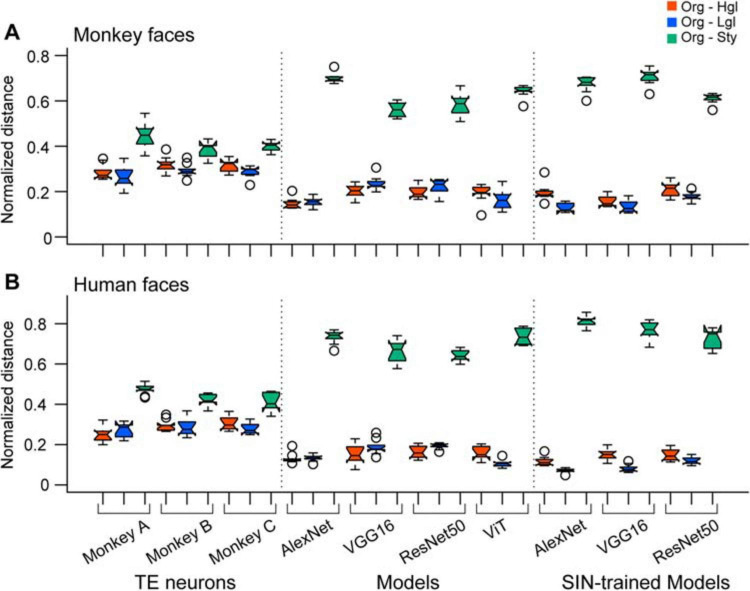
Normalized Euclidean distance between the original and surface-quality-modified face images. **(A)** Normalized Euclidean distance between the original and surface-quality-modified images for the monkey face images. **(B)** Normalized Euclidean distance between the original and surface-quality-modified images for the human face images. Org - Hgl, distance between the original and high-gloss images in orange; Org - Lgl, distance between the original and low-gloss images in blue; Org - Sty, distance between the original and style-transferred images in green. In box plots, the middle line indicates the median. The notches indicate the 95% confidence interval for the median. The whiskers extend to the most extreme data point which is no more than 1.5 times the inter-quartile range from the box.

Because the style-transferred faces were separated from or dissimilar to the original, high-gloss and low-gloss faces in both TE neurons and the models (Figure 2), we calculated the normalized Euclidean distance between population-activity/feature vectors for each original surface-quality-modified face pairs to quantitatively compare the distances (Methods, Figure 3). Normalized distances for the original–style-transferred pairs (Org – Sty in green) were consistently larger than those for the original–high-gloss (Org – Hgl in orange) and original–low-gloss (Org – Lgl in blue) pairs across all TE neurons and models, including SIN-trained models, showing greater separation of style-transferred faces from the original faces. The normalized distances for the original–style-transferred pairs were much larger in models and SIN-trained models than in TE neurons, indicating that style-transferred faces were more strongly separated from the original faces in these models than in TE neurons.

### 3.3 Centroids distance between categories

To quantitatively compare the effect of expression, identity, and surface quality on the representational space, the images of monkey and human faces were divided into three grouping schemes: expression groups, identity groups, and surface quality groups, and the averaged centroid distance between a pair of members in each group was calculated. For 3-surface quality condition (original, high-gloss, and low- gloss), a total of 27 images (3 expressions × 3 identities × 3 surface qualities) were included, while for the 4-surface quality condition (adding style-transferred), a total of 36 images (3 expressions × 3 identities × 4 surface qualities) were included. Since the centroid distances across different neural populations and models cannot be directly compared, we normalized the sum of centroid distances for the expression, identity, and surface quality to 1 for each monkey/model and analyzed their relative ratios.

**FIGURE 4:**
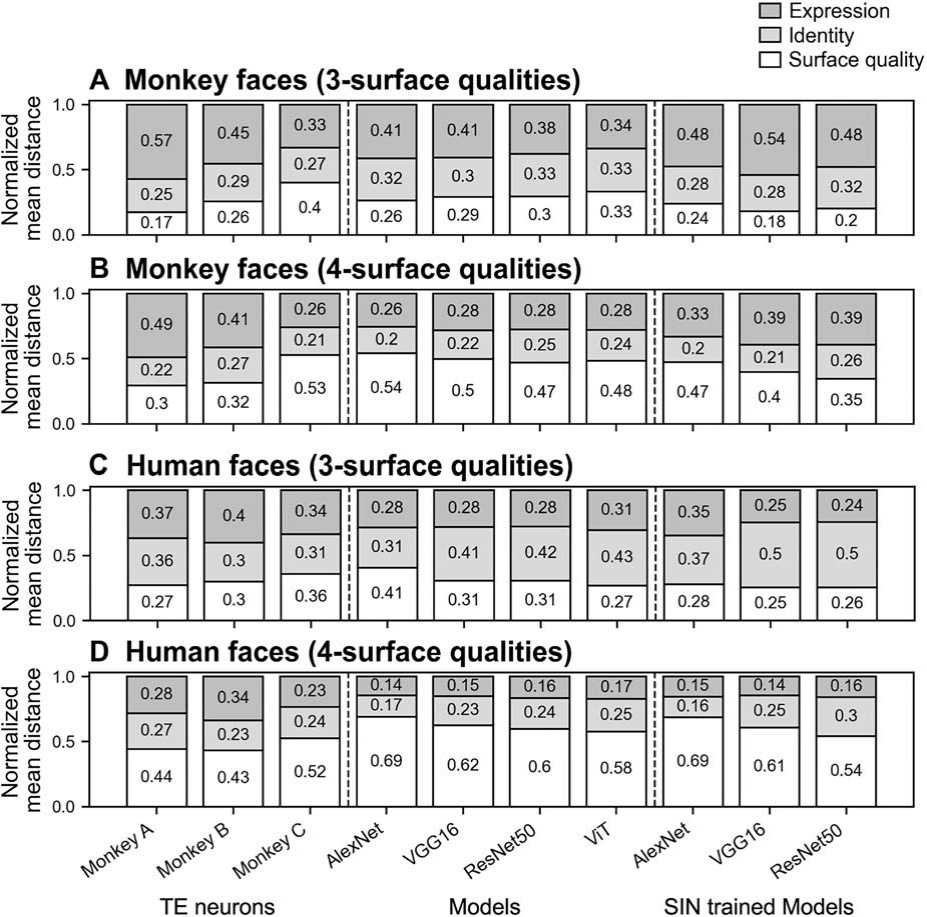
Centroid distances of each category in the representation of TE neurons and models. **(A)** Normalized average centroid distances between groups for 3-surface quality condition (original, high-gloss, and low-gloss) using monkey facial images. **(B)** Normalized average centroid distances between groups for 4-surface quality condition (original, high-gloss, low-gloss, and style-transferred) using monkey facial images. **(C)** Normalized average centroid distances between groups for 3-surface quality condition using human facial images. **(D)** Normalized average centroid distances between groups for 4-surface quality condition using human facial images.

For TE neurons, across the 3-surface quality condition (original, high-gloss, low-gloss) (Figure 4A), Monkeys A and B showed relatively small contributions of surface quality (0.17 and 0.26 for Monkeys A and B, respectively) and larger contributions of expression (0.57 and 0.45 for Monkeys A and B, respectively). Monkey C exhibited a larger contribution of surface quality (0.40), consistent with the observation in its t-SNE-based representational space. A similar pattern was observed for human-face stimuli (Figure 4C).

When style-transferred images were included (4-surface quality condition), the contribution of surface quality increased in TE neurons. For monkey-face stimuli (Figure 4B), Monkeys A and B continued to show expression as the largest component, although surface-quality contributions increased relative to the three-surface condition (e.g., Monkey A: 0.17→0.30; Monkey B: 0.26→0.32) (Figure 4B). For human-face stimuli (Figure 4D), surface quality showed the largest contributing factor in Monkeys A (0.44) and B (0.43) as well as Monkey C (0.52). Monkey C consistently showed the largest surface component across both monkey and human faces.

For model representations, the dominant component depended on both the stimulus category and the inclusion of style-transferred images. In the 3-surface quality condition for monkey-face stimuli (Figure 4A), expression showed the largest contribution in all models, including SIN-trained models. In contrast, for human-face stimuli under the 3-surface quality condition (Figure 4C), identity showed the largest contribution in most models, except for AlexNet, in which surface quality was the largest component.

For monkey-face stimuli in the 4-surface quality condition (Figure 4B), surface quality was the largest component in all ImageNet-trained models. This pattern differed from TE neurons in Monkeys A and B, in which expression remained the largest component even under the 4-surface quality condition. In the SIN-trained models, surface-quality contributions were generally reduced compared with the corresponding ImageNet-trained models, but surface quality remained the largest component in AlexNet and VGG16. In SIN trained ResNet50, however, expression (0.39) exceeded surface quality (0.35), indicating that the change in training dataset altered the relative weighting of information in this model.

For human-face stimuli in the 4-surface quality condition (Figure 4D), surface quality was the largest component in all models, including the SIN-trained models. This pattern was consistent with the TE results, in which surface quality was also the largest component in all three monkeys. However, the surface-quality contribution in every model was larger than that in Monkey C, which showed the strongest surface-quality contribution among the TE populations (Monkey C: 0.52; AlexNet: 0.69; VGG16: 0.62; ResNet50: 0.60; ViT: 0.58; SIN trained AlexNet: 0.69; SIN trained VGG16: 0.61; SIN trained ResNet50: 0.54). Thus, although training on SIN reduced the influence of surface quality in some conditions, surface quality remained a major organizing factor in model representations, particularly when style-transferred human-face images were included.

### 3.4 Image analysis

To investigate why style-transferred images are distinct from other surface quality images, we analyzed their low-level visual characteristics. Table 1 shows the median and interquartile range (Q1–Q3) of low-level visual features across different categories (Monkey, Human, pixel-randomized shapes) and surface qualities (original, high-gloss, low- gloss, style-transferred). Style-transferred images consistently exhibited higher luminance, lower saturation, and higher value compared to original, high-gloss, and low-gloss images.

**TABLE 1:** Low-level visual features of the test stimuli.

Style/metric	Monkey	Human	Pixel-randomized shape (ellipse)	Pixel-randomized shape (trapezoid)
Luminance				
Original	66.1 (48.2–109.8)	119.7 (82.2–150.1)	90.1 (84.1–96.1)	116.1 (110.9–121.3)
High-gloss	64.4 (47.4–105.0)	123.3 (82.7–164.6)	95.8 (88.8–103.0)	124.4 (118.4–130.5)
Low-gloss	67.4 (48.7–108.4)	116.3 (81.7–136.2)	90.1 (85.7–94.6)	116.2 (112.4–119.9)
Style-transferred	128.6 (98.0–185.0)	139.1 (103.4–190.7)	145.0 (138.9–151.0)	147.9 (142.4–153.3)
Hue (°)				
Original	32 (18–60)	22 (16–30)	12 (8–16)	20 (20–22)
High-gloss	32 (18–60)	24 (18–32)	12 (8–16)	20 (20–22)
Low-gloss	32 (16–226)	22 (16–30)	12 (10–14)	20 (20–22)
Style-transferred	32 (18–90)	24 (18–34)	12 (10–16)	22 (20–22)
Saturation (%)				
Original	31.4 (24.3–40.0)	36.9 (31.4–41.2)	22.4 (20.4–24.3)	33.7 (32.5–35.3)
High-gloss	30.6 (22.0–39.6)	34.9 (27.8–40.4)	20.8 (19.2–22.7)	31.8 (30.6–32.9)
Low-gloss	30.6 (23.9–39.2)	38.4 (32.5–42.7)	22.0 (20.8–23.5)	33.7 (32.5–34.5)
Style-transferred	16.9 (11.8–23.5)	27.5 (18.4–38.0)	13.3 (12.2–14.5)	25.9 (24.3–27.1)
Value (%)				
Original	29.0 (21.6–44.3)	58.4 (38.0–74.5)	41.2 (38.8–43.9)	55.7 (53.3–58.4)
High-gloss	29.0 (21.6–45.1)	56.9 (35.7–83.5)	43.5 (40.4–46.7)	59.2 (56.1–62.0)
Low-gloss	31.4 (22.7–52.2)	58.0 (38.8–69.4)	41.2 (39.2–43.1)	55.7 (53.7–57.6)
Style-transferred	52.5 (41.2–84.7)	61.2 (45.9–91.8)	62.0 (59.6–64.7)	67.1 (64.7–69.4)

Median and interquartile range (Q1–Q3) of low-level visual features (Luminance, Hue, Saturation, Value) across different species (monkey, human, ellipse, trapezoid) and surface qualities (original, high-gloss, low-gloss, style-transferred). For monkey and human images, the statistics were calculated using nine images, combining all expressions and identities. In contrast, for ellipse and trapezoid, the statistics were computed using a single image for each. Luminance values were computed from RGB images by converting each pixel to grayscale intensity using the standard ITU-R BT.601 luma transform (*Y* = 0.299R + 0.587G + 0.114B), implemented in Python with the Pillow library (Image.convert(“L”)). Hue, Saturation, and Value were obtained by converting RGB images to the HSV color space using OpenCV (cv2.cvtColor(…, cv2.COLOR_RGB2HSV)). To match standard conventions, Hue was expressed in degrees (0–360°) and Saturation and Value were normalized to percentages (0%–100%).

Although style transfer alters the low-level features (Table 1), the distinct separation observed for style-transferred face images cannot be explained by these features alone, because the neuronal population vectors for pixel-randomized shapes clustered together, regardless of the surface qualities from which the shapes were derived (Figure 2A). Instead, the effect likely reflects higher-order processing of facial information, beyond simple pixel-level properties.

## 4 Discussion

We examined how neuronal activity in macaque area TE represents face images in terms of facial features (expression and identity) and surface qualities (gloss and style transfer), and compared the neuronal representation with representation in the neural network models. Regarding the effect of surface qualities in TE neurons, activity in more neurons was affected by the style-transferred face images than by gloss-modulated faces (Figure 1D), indicating a strong influence of style transfer on neuronal activity. In both the representation by TE neurons and those by the models, the style-transferred faces were represented separately from the remaining faces and shapes (Figure 2A), demonstrating a consistent pattern across biological and artificial systems. The separation between the original–style-transferred face pairs was larger than that between the original–gloss-modulated face pairs, and this difference was more pronounced in the models than in TE neurons (Figure 3), suggesting that the style transfer had a stronger impact on model representations.

In addition to the effect of style transfer, differences between TE neurons and the models were observed in the representation of facial features and surface qualities. Whereas most of the models that were tested in this study exhibited relatively consistent representations, TE neurons showed a degree of diversity across subjects (Figures 2, 4) (Monkey A: representation of monkey vs. human vs. shape categorization and monkey facial expression; Monkey B: monkey vs. human vs. shape categorization; and Monkey C: face vs. shape categorization and facial gloss modulation). This pattern was also reflected in the centroid-distance analysis (Figures 4A,B), in which Monkeys A and B showed larger contributions of expression for monkey faces than surface quality, whereas Monkey C showed the opposite tendency.

This across-subject heterogeneity may reflect differences in the recording sites or local connectivity within TE rather than individual variation *per se*. The recording sites for Monkeys A and B were located in anterior TE: in STSv for Monkey A (Supplementary Figure 1), and between the STS and AMTS for Monkey B (Supplementary Figure 1). Recordings in Monkey C were obtained from posterior TE, just anterior to PMTS (Supplementary Figure 1). Thus, posterior TE may be more involved in surface information processing, whereas anterior TE may be more related to shape- and face-feature, including facial expression, processing. Consistent with this interpretation, previous studies have reported that surface gloss of objects is represented in posterior TE (Nishio et al., 2012, 2014), and that facial expression is represented in anterior TE including STSv (Morin et al., 2015; Sugase-Miyamoto et al., 2014). Furthermore, Okazawa et al. (2012) showed, using fMRI, that within central IT–approximately equivalent to posterior TE–there was partial overlap between regions activated by face images and those activated by specular reflection of objects, suggesting the existence of neurons responsive to both faces and surface quality. Jagadeesh and Livingstone (2024) recently reported a tendency for IT neurons to prioritize texture over shape using object images with different textures, suggesting a texture bias in the ventral visual pathway. Our results refine this view for TE: in addition to the rather uniform bias of style transfer, TE showed multiple representational patterns–expression, species, and gloss–which likely reflect differences in the recording locations within TE.

Neural network models, including AlexNet, VGG16, ResNet50, and ViT, showed larger contributions of the surface qualities than contributions of the facial expression or identity in the 4-surface quality condition compared to the 3-surface quality condition, particularly for human-face stimuli (Figures 4B,D compared to Figures 4A,C). This tendency aligns with previous findings from Geirhos et al. (2018) and Tuli et al. (2021), which demonstrated that CNNs and ViTs tend to emphasize surface qualities over shapes when these features conflict upon identification of image contents. Even in models trained on SIN, surface-quality contributions remained large in the 4-surface quality condition, although SIN training reduced or altered this tendency in some cases. In Geirhos et al. (2018), although models trained on SIN exhibited reduced texture dependence compared to those trained on standard ImageNet, they still showed a higher degree of texture dependence than human observers. Therefore, our results are consistent with and extend these previous findings. Specifically, while texture bias can be reduced through training data, it is not eliminated, suggesting that this tendency cannot be explained solely by the characteristics of the training data. Tuli et al. (2021) found that ViTs have less texture bias compared to CNNs when dealing with images that conflict between texture and shape. However, in our study, we found that in the final layer, ViT still emphasized surface quality over expressions and identities to a similar extent as CNNs, when compared to TE neurons (Figure 4).

Shi et al. (2024) reported that CNNs exhibit greater robustness to silhouette images than ViTs, likely because CNNs can classify based on contour information alone, hypothesizing that this robustness may be attributed to the larger receptive field sizes of CNNs. Therefore, analyzing how receptive field sizes contribute to surface quality processing across monkey TE, CNNs, and ViTs using gloss-modulated and silhouette images could provide further insights into model and neuron sensitivities to surface quality features. Previous studies suggested that the receptive field size of neurons varies within TE (Op De Beeck and Vogels, 2000) and that neurons in anterior face patches within anterior TE are tuned to local facial features rather than feature combinations (Waidmann et al., 2022). Such variation may account for the observed differences, with neurons that are more strongly influenced by the surface quality potentially having smaller receptive fields. In CNNs, reducing kernel size leads to a greater emphasis on fine-scale surface details, whereas increasing kernel size allows for more global shape feature extraction. Indeed, it has been shown that increasing CNN kernel size reduces texture bias–the tendency to prioritize surface quality over shape (Ding et al., 2022). Thus, the coexistence of neurons that prioritize surface quality and those that prioritize shape may reflect differences in receptive field sizes within TE.

Although the present analyses characterize the representational geometry of TE and model responses, they do not directly test how readily expression, identity, or surface-quality information can be decoded; such readout-based analyses will require larger stimulus sets and remain an important direction for future work.

## Data Availability

The raw data supporting the conclusions of this article will be made available by the authors, without undue reservation.

## Footnotes

1
https://www.jnss.org/en/animal_primates

2
https://unit.aist.go.jp/hiiri/nstrg/download/iRecHS_Site/index_j.html

3
https://github.com/rgeirhos/Stylized-ImageNet
