# Supplementary material for: Representations of facial features and surface quality in monkey area TE compared to neural network models
Source: Front Neural Circuits. 2026 Aug 3;20:1783892. doi: 10.3389/fncir.2026.1783892 (PMC13478094; doi:10.3389/fncir.2026.1783892)
Supplement: Supplementary file 1 [file Table_1.docx]

Supplementary Material

# 1 Supplementary Figure

**Supplementary Figure 1.** Recording sites. (*A*): MRI showing recording sites (an open broken rectangle at the ventral bank of STS) in Monkey A. The sites were located at 22 mm rostral (A22) to the interaural line. MRIs were obtained on a Philips Ingenia 3 T MRI system, using a flexible surface coil and a 3-dimensional T1 weighted imaging (repetition time/echo time =7.3/3.2 ms, flip angle = 8, voxel size =0.6 ×0.6 ×0.6 mm3/voxel, matrix size =224 ×224 ×200). (*B* and *C*): Utah array locations in Monkeys B and C, respectively. amts, anterior middle temporal sulcus; ots, occipital temporal sulcus; pmts, posterior middle temporal sulcus; sts, superior temporal sulcus; anterior TE, the anterior part of TE; posterior TE, the posterior part of TE; STSv, the ventral bank of STS.
